# *In Vivo* Fluorescence Immunohistochemistry: Localization of Fluorescently Labeled Cetuximab in Squamous Cell Carcinomas

**DOI:** 10.1038/srep10169

**Published:** 2015-06-29

**Authors:** Esther de Boer, Jason M. Warram, Matthew D. Tucker, Yolanda E. Hartman, Lindsay S. Moore, Johannes S. de Jong, Thomas K. Chung, Melissa L. Korb, Kurt R. Zinn, Gooitzen M. van Dam, Eben L. Rosenthal, Margaret S. Brandwein-Gensler

**Affiliations:** 1 Division of Otolaryngology, University of Alabama at Birmingham, USA; 2 Department of Surgery, University Medical Center Groningen, University of Groningen, The Netherlands; 3 Department of Pathology, University Medical Center Utrecht, University of Utrecht, Utrecht, The Netherlands; 4 Division of Advanced Medical Imaging, Department of Radiology, University of Alabama at Birmingham, Birmingham, AL, USA; 5 Department of Surgery, Nuclear Medicine and Molecular Imaging and Intensive Care, University Medical Center Groningen, University of Groningen, The Netherlands; 6 Division of Anatomic Pathology, Department of Pathology, University of Alabama at Birmingham, USA

## Abstract

Anti-EGFR (epidermal growth factor receptor) antibody based treatment strategies have been successfully implemented in head and neck squamous cell carcinoma (HNSCC). Unfortunately, predicting an accurate and reliable therapeutic response remains a challenge on a per-patient basis. Although significant efforts have been invested in understanding EGFR-mediated changes in cell signaling related to treatment efficacy, the delivery and histological localization in (peri-)tumoral compartments of antibody-based therapeutics in human tumors is poorly understood nor ever made visible. In this first in-human study of a systemically administered near-infrared (NIR) fluorescently labeled therapeutic antibody, cetuximab-IRDye800CW (2.5 mg/m^2^, 25 mg/m^2^, and 62.5 mg/m^2^), we show that by optical molecular imaging (i.e. denominated as *In vivo* Fluorescence Immunohistochemistry) we were able to evaluate localization of fluorescently labeled cetuximab. Clearly, optical molecular imaging with fluorescently labeled antibodies correlating morphological (peri-)tumoral characteristics to levels of antibody delivery, may improve treatment paradigms based on understanding true tumoral antibody delivery.

The application of antibody-based therapies has significantly grown in the past 15 years; it is currently one of the most important targeted treatment strategies for hematological and solid malignancies^1^. Recently, cancer immunotherapy was announced to be the breakthrough of the year 2013. Targeting specific signaling pathways or immune effector functions has a number of advantages over conventional cytotoxic chemotherapeutics, such as reduced systemic off target effects and more specific cell killing^2^. Furthermore, antibody-based therapies can also be rationally combined with conventional chemotherapy to synergistically enhance cell killing with non-overlapping toxicities^3^^,^^4^. The development of anti-EGFR (epidermal growth factor receptor) strategies has been one of the most successful targeted therapies, especially for head and neck squamous cell carcinoma (HNSCC)^5^. Cetuximab is an FDA approved anti-EGFR human-murine chimeric monoclonal antibody (mAb) that is currently used in combination with radiotherapy in the treatment of HNSCC, or as a single agent in patients with recurrent/metastatic disease for whom prior platinum-based therapy has failed^6^.

However, only 10–13% of patients with HNSCC are expected to benefit from EGFR inhibitors due to occurrence of reactivation downstream signaling despite the presence of the EGFR inhibitor, and predicting a patient response in terms of complete, partial or no (pathological) response remains challenging^7^. Appropriate patient selection would limit the considerable costs and significant morbidity associated with unnecessary delivery of antibody based therapeutics, and thus overtreatment without the target of choice being present. EGFR inhibitors block ligand-binding, abrogating activation of downstream tumor-promoting pathways including apoptosis evasion (PI3K-Akt), proliferation (Ras-Raf-MEK-MAPK), and transcription (STAT3)^8^. Constitutive activation of STAT3 is considered the predominant promoter of EGFR inhibitor resistance^9^. However interrogation of these pathways in human disease has not led to reliable prediction of patients likely to respond to therapy. Although there have been substantial efforts to evaluate relationships between various molecular markers (such as levels of EGFR and angiogenesis) and clinical outcome by using diagnostic biopsy materials, these efforts have not been successful, which is thought to be in part due to the variability in tissue penetration and delivery of these large proteins of ~150 kD^10^.

Because of difficulties in quantitating distribution and penetration of antibody-based therapeutics in human solid tumors, there is limited understanding of peri- and intratumoral delivery, and how this might impact patient response, together with local and systemic toxicity. In this study, we evaluate, for the first time in humans, the distribution of intratumoral anti-EGFR antibody using cetuximab conjugated to the near-infrared fluorescent dye IRDye800CW (cetuximab-IRDye800CW), which does not alter the binding capacity of cetuximab, pharmacokinetics or biodistribution, as previously shown^11^. Correlating tumor and stromal characteristics with fluorescence labeled antibody delivery to the tumor may serve as an opportunity to provide additional information about predictors of clinical response and toxicity to therapeutic antibodies, but also to resistance patterns by labeling other effector proteins with different fluorescent wavelengths providing multispectral (NIR) antibody and effector imaging *in situ*. To this end, we evaluated systemically administered cetuximab-IRDye800CW and its *in situ* fluorescence as a correlate of antibody distribution in surgical resection specimens, in the context of a Phase I safety and toxicity trial in patients with HNSCC (Clinicaltrials.gov Identifier: NCT01987375). We tested the hypothesis that EGFR density, vascular density, and tumor proliferation are directly associated with tumor cetuximab-IRDye800CW fluorescence localization and intensity.

## Results

**Figure 1:**
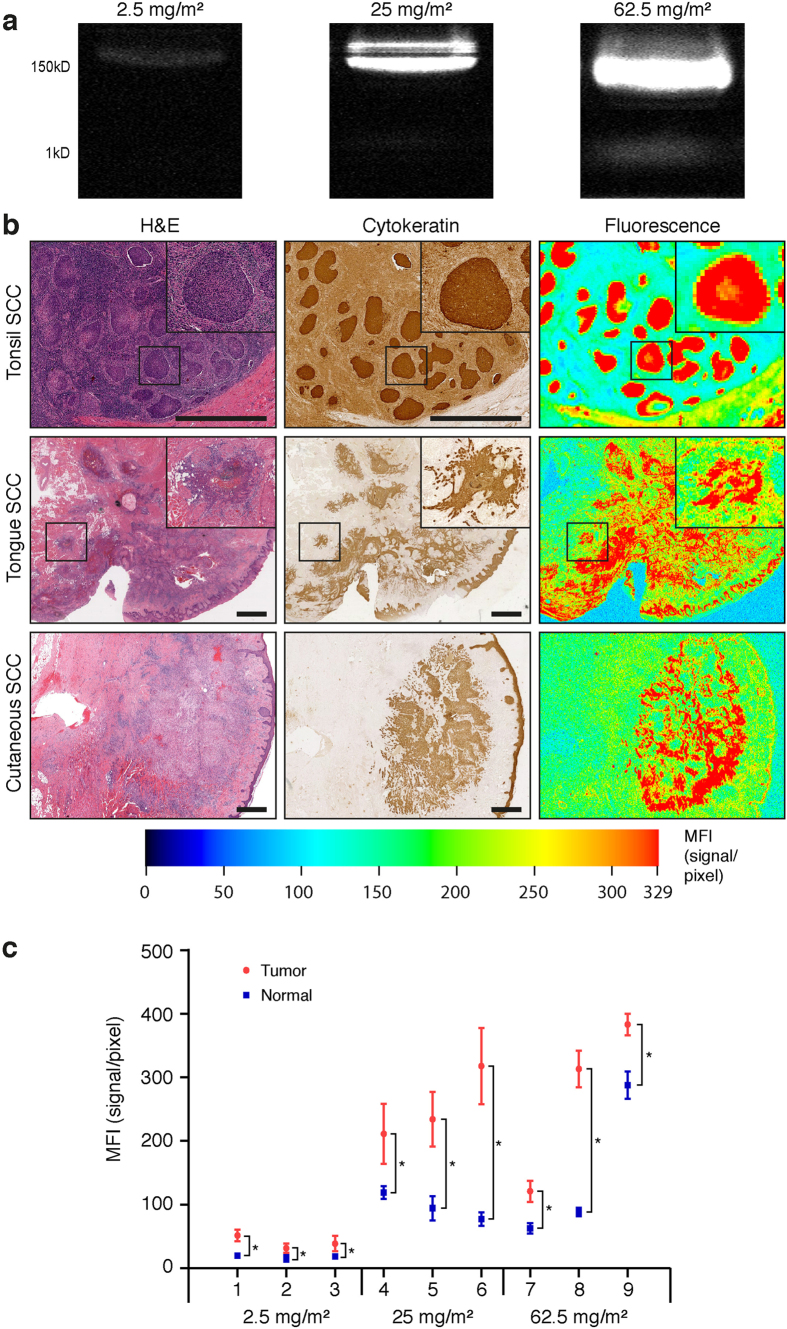
Localization of cetuximab-IRDye800CW in histologic sections of tissue with tonsil, tongue and cutaneous squamous cell carcinoma (SCC). (**a**) Representative 150 kD (=intact cetuximab-IRDye800CW) and 1kD (=IRDye800CW) bands are shown by SDS-PAGE of individual patient blood samples of each dosage cohort. (**b**) Representative haemotoxylin/eosin (H&E), cytokeratin and fluorescence image of SCC sections of tonsil, tongue and cutaneous origin. (**c**) Mean Fluorescence Intensity (MFI) quantified in pathology-positive areas of tumor and healthy surrounding for nine patients (n = 3 at each dosage cohort) for respectively 2.5 mg/m^2^, 25 mg/m^2^, and 62.5 mg/m^2^. Data are presented as mean ± SD, ^*^ = *P* < 0,001. Scale bars in all images represent 100 μm.

There were a total of 9 patients with advanced squamous cell carcinoma arising in the head and neck that were systemically injected with cetuximab-IRDye800CW prior to curative surgical resection of their tumor. To confirm the integrity of the antibody-dye conjugate after systemic injection, SDS-PAGE of blood samples by imaging gels at the near infrared range demonstrates fluorescence bands at the expected molecular weight of 150kD while free, unbound dye could not be detected (Fig. 1a). A toxicology study performed in non-human primates has demonstrated that cetuximab-IRDye800CW has the same serum half-life as cetuximab (i.e. 2.5 days) and retains similar antigen binding specificity^11^. Our study demonstrated the cetuximab-IRDye800CW serum half-life to be 1.2 days, 1.3 days and 1.6 days for the 2.5 mg/m^2^, 25 mg/m^2^ and 6^2^.5 mg/m^2^ doses, respectively. Surgical pathology specimens of the tumor and normal tissues were imaged using a closed field imaging system (Odyssey, LI-COR Biosciences) prior to histological sectioning and the differential fluorescence measured (Fig. 1b). Cetuximab-IRDye800CW localized specifically in tumor regions, as confirmed by histology and cytokeratin immunohistochemistry. Analysis of the histological sections with equal thresh holding demonstrated that the mean fluorescence intensity (MFI) was significantly higher in SCC compared to corresponding adjacent tissues (*P* < 0.001) across all three dosing cohorts (Fig. 1c). The tumor-to**-**normal-ratio of cetuximab-IRDye800CW MFI was 2.2, 2.5, and 1.9 at 2.5 mg/m^2^, 25 mg/m^2^ and 62.5 mg/m^2^ doses, respectively. A 10-fold increase in single infusion dose (25 mg/m^2^ vs. 2.5 mg/m^2^) resulted in an almost 7-fold increase in MFI. Yet, further 25-fold increase in infusion dose (2.5 mg/m^2^ vs. 62.5 mg/m^2^) yielded only 8.8-fold increase in the MFI, with decreased tumor/normal ratio due to higher background (i.e. from 2.5 to 1.9).

### Molecular correlates

Using fluorescence intensity as a readout for cetuximab-IRDye800CW accumulation within the tissues, the MFI was correlated with biological characteristics of the tumor that might also influence antibody (peri-)tumoral distribution and binding into the tumor including cytokeratin (tumor density), EGFR expression, Factor VIII (vascular density) and Ki67 (proliferation) for each dose group (Table 1). Univariate analysis with respect to SCC demonstrated that tumor density, EGFR expression, and vascular density were directly associated with MFI (*P* < 0.05) in all three dosing cohorts, whereas Ki67 and MFI were significantly associated (*P* < 0.05) at the lowest dose concentration group (2.5 mg/m^2^). EGFR expression was independently associated with MFI, when adjusted for cytokeratin, Factor VIII and Ki67, at doses of 2.5 mg/m^2^ and 25 mg/m^2^, (*P* < 0.001).

### EGFR fluorescence immunohistochemistry

**Figure 2:**
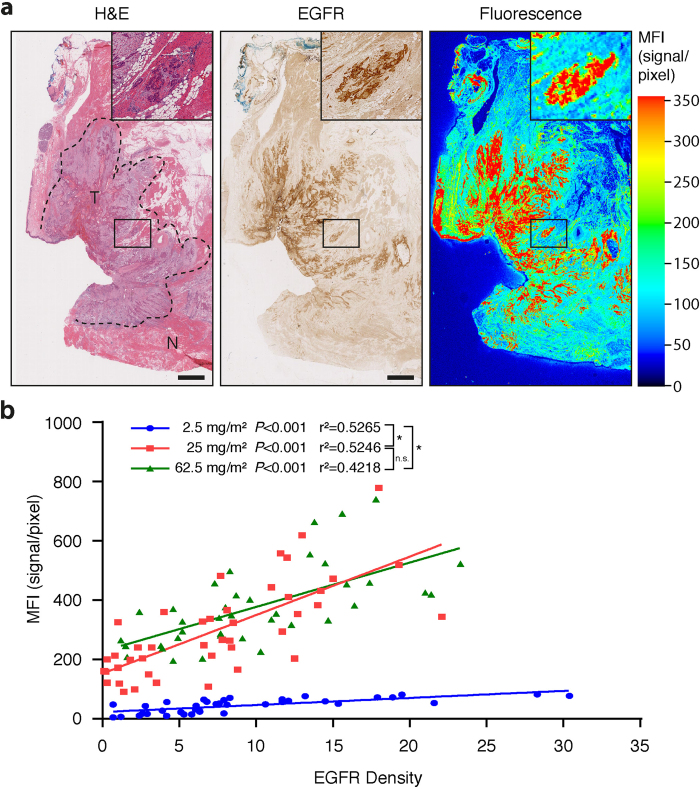
Co-localization of fluorescence signals of cetuximab-IRDye800CW and Epidermal Growth Factor Receptor (EGFR) expression. (**a**) Representative haemotoxylin/eosin (H&E) image indicating tumor (T) and normal (N) with corresponding EGFR expression immunohistochemistry stain and fluorescence image. (**b**) Increase in Mean Fluorescence Intensity (MFI) as a function of EGFR Density (% area EGFR positive). Data are presented as mean ± SD, * = P < 0,001. Scale bars in all images represent 100 μm.

Further analysis to correlate EGFR expression with MFI showed a strong correlation of squamous cell carcinoma (SCC) with EGFR expression (Fig. 2a). Clearly, cetuximab-IRDye800CW uptake within SCC was specifically localized to areas that expressed EGFR. The univariate association between EGFR density and MFI was observed at all three dosing cohorts (*P* < 0,001, Fig. 2b). A 6-fold increase in MFI was seen at the two higher doses compared to the lower dose concentration (*P* < 0,001); no significant increase in MFI was seen when comparing the highest dose (62.5 mg/m^2^) with the middle dose 25 mg/m^2^ (*P* > 0.05).

### Fluorescence intensity correlates with distance to tumor

**Figure 3:**
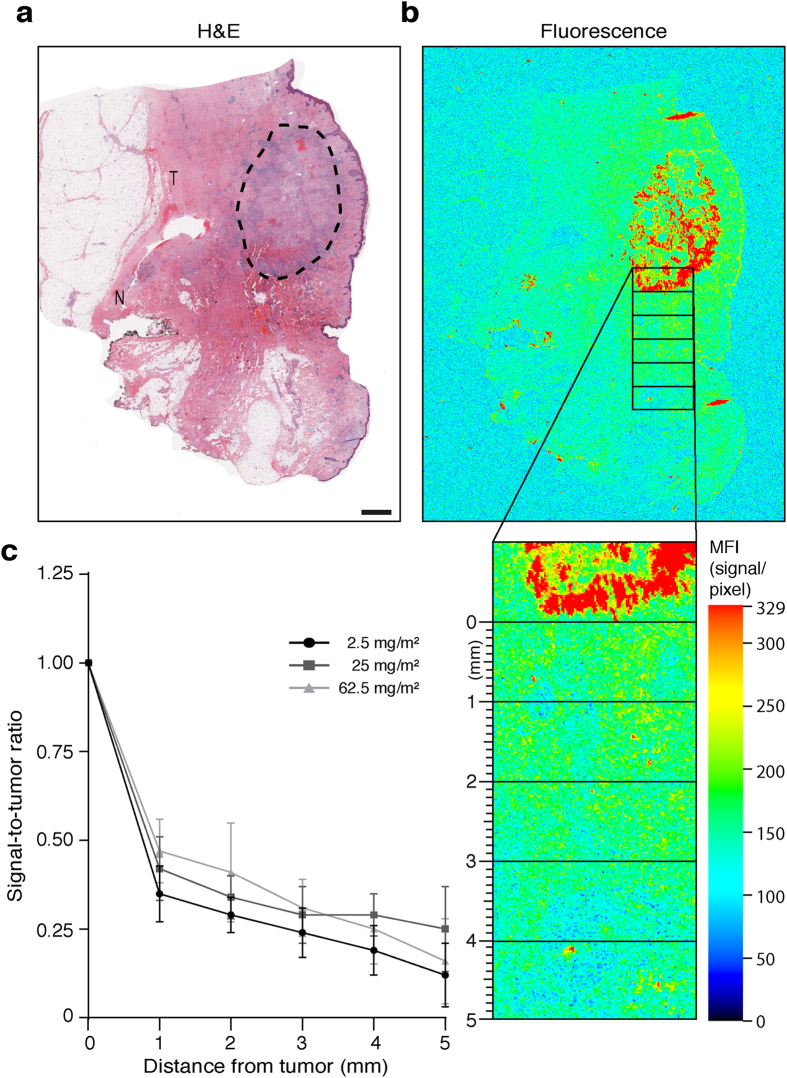
Fluorescence from adjacent normal tissue correlates with distance from tumor.Representative H&E image tumor (T) and normal (N) regions outlined by a board-certified pathologist (a). Signal-to-tumor ratio is determined at predefined distances from tumor (1-5 mm) as depicted by rectangular regions-of-interest (ROI) at 0, 1, 2, 3, 4 and 5 mm margin distance (b) graphed (c). Data are presented as mean ± SD. Scale bar represent 50 μm.

Representative H&E with tumor (T) and normal (N) are outlined in Fig. 3a and the corresponding tissue slide quantified in the fluorescence image (Fig. 3b). As demonstrated in Fig. 3c, a significant correlation between distance from tumor and signal-to-tumor ratio was shown (P < 0.001, P = 0.02 and P < 0.001; R square 0.996, 0.891 and 0.994 for the 2.5, 25 and 62.5 mg/m2 dosing cohorts, respectively) throughout all three dosing cohorts. A significant decrease in MFI (P < 0.001; 2.9, 2.4 and 2.1-fold reduction for the 2.5, 25 and 62.5 mg/m2 dosing cohorts, respectively) was shown at a distance of 1 mm from the tumor. Importantly, MFI significantly decreased when comparing the values between 5 mm and 1 mm from the tumor edge (P < 0.001; 8.3, 4.0 and 6.3-fold reduction for the 2.5, 25 and 62.5 mg/m2 dosing cohorts, respectively).

### Intratumoral cetuximab-resistant areas

**Figure 4:**
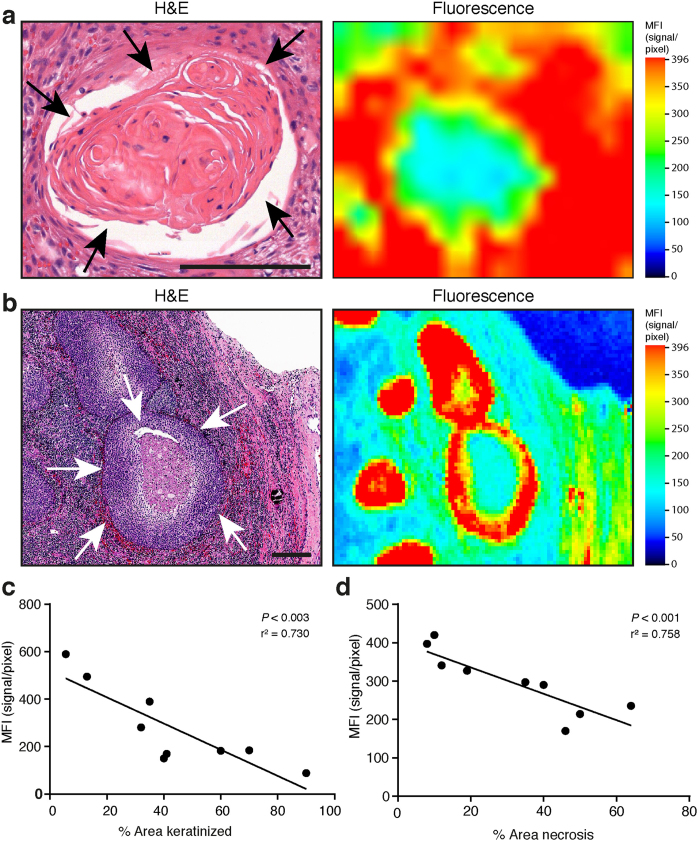
Effect of tissue properties on cetuximab-IRDye800CW uptake. (**a**) Representative haemotoxylin/eosin (H&E) image and corresponding fluorescence image. Differentiated keratinizing cancer cells are outlined by black arrows. (**b**) White arrows outline tumor necrosis. (**c**) Regression analysis between % Area keratinized and Mean Fluorescence Intensity (MFI) (*P* < 0.003). (**d**) Regression analysis between % Area necrosis and Mean Fluorescence Intensity (MFI). Data are presented as mean ± SD, ^*^ = *P* < 0,001. Scale bars in all images represent 100 μm.

Despite overall strong correlation between EGFR and SCC, there were discordant areas within tumors with high EGFR-expression that demonstrated low fluorescence. Histologically, these areas correspond to mature, differentiated keratinizing cancer cells that expressed moderate levels of EGFR, yet did not incorporate the antibody-dye conjugate (Fig. 4a). Comparing MFI of mature, differentiated keratinizing cancer cells to non-differentiated cancer area show enormous reduction in MFI, despite equal EGFR expression levels (Supplementary Fig. 1). Additionally, tumor necrosis was also clearly associated with areas of low fluorescence (Fig. 4b). Regression analysis collapsing the two groups with middle and highest doses (25 mg/m^2^ and 62.5 mg/m^2^) revealed an inverse relationship between cancer keratinization (Fig. 4c), and tumor necrosis (Fig. 4d) with MFI (*P* = 0.003 and *P* < 0.001 respectively). SCC with ≥ 50% keratinization, or ≥ 50% necrosis demonstrated a larger than 2-fold, and 1.7-fold decreased MFI.

### Extratumoral ***in vivo*** fluorescence immunohistochemistry

EGFR is expressed in a wide range of normal tissues and anti-EGFR antibody targeting of these tissues influences the dose-dependent toxicities of therapy. In epidermal tissues, EGFR expression and corresponding cetuximab-IRDye800CW fluorescence was observed on the cell membrane of the basal cells and in the superficial cells undergoing terminal differentiation (Supplementary Fig. 2). Conversely, limited cetuximab-IRDye800CW uptake was observed in the more superficial differentiated epithelium. Sebaceous glands exhibited areas of high EGFR expression, which correlated with increased localization of cetuximab-IRDye800CW (Supplementary Fig. 2).

## Discussion

Although anti-EGFR therapies have been in clinical use for almost a decade in patients with squamous cell carcinoma, there are no biomarkers that successfully predict patient response to treatment. Furthermore, there is minimal understanding of antibody penetration within the heterogeneous histological intratumoral and peritumoral compartments. In the first application of this technology in humans, we show that *in vivo fluorescence immunohistochemistry* (systemic injection of a near-infrared fluorescent monoclonal therapeutic antibody) can be used to localize cetuximab-IRDye800CW. This provided evidence that cetuximab delivery correlated with the expression of EGFR in almost all of the tumor tissues excised and provided significant insight into antibody compartmentalization not previously known or observable in humans. Interestingly, well-differentiated keratinizing tumor cells expressed high levels of EGFR by immunohistochemistry, yet had significantly lower local fluorescence. This suggests that well-differentiated, keratinizing regions within squamous cell carcinomas may not be as effectively targeted with anti-EGFR therapies, although downstream effector proteins thought to be responsible for resistance, such as constitutive activation of STAT3, are not yet possible to track down *in vivo*. Other molecular markers that have been considered important for drug delivery and tumor penetration such as vascular density or tumor cell distribution surrounding (neo-)vasculature was not demonstrated in this cohort of nine patients and does not support the Enhanced Permeability and Retention (EPR) effect to be responsible for the distribution but more towards target-specific binding of cetuximab-IRDye800CW, as shown previously^11^.

Cetuximab-IRDye800CW specifically localized to SCC tumor cells arising in the head and neck and does not accumulate in non-epithelial tissues. Significantly higher mean fluorescent intensities (MFIs) were consistently noted within the carcinomas compared with normal tissues in all three dosing cohorts. When using a diagnostic dose of cetuximab, rather than repeated therapeutic delivery, higher cetuximab dosing may not correspond to greater cetuximab delivery in tumor tissues. Instead, more off-target uptake may be expected with less intratumoral uptake due to receptor saturation at the highest dose, as demonstrated using escalating doses of cetuximab-IRDye800CW.

We demonstrated that fluorescence was strongly correlated with the location of tumor and fell precipitously at a distance of 1 mm from the tumor edge (*P* < *0.001*) in MFI intensity. This very sharp demarcation between tumor and normal adjacent tissue was consistently measured throughout all three dosing cohorts. *In vivo* fluorescence immunohistochemistry could be used during frozen section analysis as a part of intraoperative fluorescence guided pathology, so that the surgeon could determine whether they are at an optimal distance.

We used *in vivo* fluorescence immunohistochemistry to provide insight into the mechanisms determining antibody delivery in SCC. To this end, we evaluated a number of biomarkers which are thought to modulate drug delivery or and peri- and intratumoral distribution. It was not surprising that fluorescence intensity correlated significantly with EGFR expression density, since cetuximab-IRDye800CW accumulation depends upon EGFR expression as shown earlier^11^. However, earlier reports have mentioned that EGFR expression level is a poor predictor of response to cetuximab^12^. While we did not assess therapeutic response, we did observe that EGFR expression strongly correlated with overall fluorescence intensity, however, EGFR expression was associated with discordantly lower fluorescence intensity in regions of well-differentiated, keratinizing SCC.

We demonstrate that in tumor areas with ≥ 50% well differentiated tumors, these areas are associated with 2-fold decreased fluorescence compared with non-keratinizing foci within the same carcinoma. While reduced uptake in well-differentiated areas within the tumor could be attributed to reduced accessibility or vascular permeability, the findings reported here are consistent with previous work demonstrating that EGFR loses ligand-binding affinity during maturation and differentiation^13^^,^^14^. We hypothesize that tumor maturation and keratinization contributes to decreased ligand affinity of EGFR or tissue penetration of the antibody, and may promote EGFR-inhibitor resistance. In addition, we also confirm tumor necrosis does not allow for cetuximab-IRDye800CW accumulation, which has also been recently noted by others in preclinical models^15^, and might be due to increased intratumoral pressure preventing influx of the cetuximab-IRDye800CW. For imaging and therapeutic purposes this might not be a limiting factor as the majority of vital tumor cells are situated around the periphery of tumor necrosis, which are of particular interest for image-guided surgery. Moreover, surgical margins are related to outgrowing vital tumor cells and not hypoxic, necrotic tumor cores.

Localization of our cetuximab-IRDye800CW bioconjugate in normal healthy tissues does explain some of the known toxicities associated with anti-EGFR therapy. Papulopustular eruption is an adverse reaction of the skin commonly seen in cancer patients treated with EGFR inhibitors^16^. The underlying mechanism, however, is still poorly defined. However, some have speculated that sebaceous glands might be the primary target of EGFR inhibitors and, as such, play a major role in the pathophysiology of papulopustular eruption^17^. Consistent with this hypothesis, we have clearly demonstrated strong fluorescence intensity in sebaceous glands. These data support the hypothesis that papulopustular eruption results from accumulation of cetuximab as can be extrapolated from the data derived by the diagnostic agent cetuximab-IRDye800CW. Significant fluorescence levels were also found in the submandibular glands and sublingual glands, which showed the same intensity levels as vital tumor cells, suggesting significant antibody targeting of the normal mucous glands. It is unclear if this results in clinically significant xerostomia since cetuximab is commonly given in conjunction with radiotherapy and as such the drug related toxicity is hard to differentiate from xerostomia caused by radiotherapy but very likely to result also by cetuximab off-site targeting.

Histological analysis of human tumors presented here suggests that the antibody-dye bioconjugate is highly specific for cancer cells and does not aspecifically accumulate within the stromal components of the tumor as might be the case when there is a profound EPR effect. The time interval between cetuximab-IRDye800CW and surgical resection was approximately 3–4 days, which provided sufficient opportunity for the agent to circulate, as shown in both animal and human immunoPET and antibody-IRDye800CW studies^18^. Moreover, the shorter half-life of cetuximab-IRDye800CW (1.2 days, 1.3 days and 1.6 days for the 2.5 mg/m^2^, 25 mg/m^2^ and 62.5 mg/m^2^ doses, respectively), compared to the original cetuximab helped to minimize background fluorescence signal.

## Conclusion

This is the first clinical study to evaluate peri- and intratumoral localization of an anti-EGFR fluorescently labeled antibody after systemic administration and denominated as *in vivo* fluorescence immunohistochemistry. Although EGFR expression correlated clearly with fluorescence intensity, further analysis of well-differentiated tumors with high levels of EGFR expression demonstrated near absent fluorescence suggesting poor uptake of the antibody in this histological subtype. As a spin-off of using therapeutic monoclonal antibodies as optical tracers in image-guided surgery, *in vivo* fluorescence immunohistochemistry provides unsurpassed information of biodistribution and correlates with standard histopathology which can be of great value in drug development of cancer immunotherapy Finally, this platform of using fluorescently labeled antibodies may be helpful to further elucidate effector proteins by multispectral fluorescent *in situ* labeling.

## Methods

### Patients and patient tissue samples

Surgical resection specimens were collected from nine consented patients enrolled in a dose escalation (2.5 mg/m^2^, 25 mg/m^2^, and 62.5 mg/m^2^) clinical trial (Clinicaltrials.gov Identifier: NCT01987375) evaluating the safety and tumor-specificity of systemically injected cetuximab-IRDye800CW for surgical navigation in patients with HNSCC. As previously reported by us, IRDye800CW was conjugated to cetuximab at a molar ratio of (dye:protein) 2.3:1^11^. Per trial design, patients received respective systemic intravenous infusion of cetuximab-IRDye800CW 3–4 days prior to the scheduled surgical procedure. Pharmacokinetics assessments were performed prior to infusion and 2 hr, 24 hr, 3-4 days, 15 days and 30 days after study drug infusion. Aliquots of plasma samples (2.5 μL) were resolved by NuPAGE 4–12% Bis-Tris gel (Invitrogen Corporation; Carlsbad, CA). Mean fluorescence intensity (MFI) from size-matched regions of interest (ROIs) was recorded. A regression line was generated to determine plasma clearance of cetuximab-IRDye800CW. Informed consent was obtained from all patients and the University of Alabama Institutional Review Board approved the study. Patient data were anonymized and all experiments using the specimens were conducted in accordance with the rules and regulations approved by the University of Alabama Institutional Review Board.

### Fluorescence detection

Study patients received preoperative systemic intravenous infusions of cetuximab-IRDye800CW. As fluorescence is fairly undiminished by histological processing, fluorescence intensity remains measurable in the pathological specimens^19^. The fluorescence intensity of IRDye800CW was measured using unstained formalin fixed paraffin-embedded on glass slides sections (5 μm) of interest with a fluorescence flatbed scanner specifically designed for imaging IRDye800CW (Odyssey, LI-COR Biosciences); slides are left unstained to avoid interference with fluorescent measurements. MFI was measured within six regions of interest (ROIs) of squamous cell carcinoma (SCC) and also healthy surrounding tissue. Sodium dodecyl sulfate polyacrylamidegel electrophoresis (SDS-PAGE) was performed on blood samples of the included patients at day 15 to ensure association between cetuximab and IRDye800CW as a complete cetuximab-IRDye800CW conjugation as described previously by us^20^.

### Sample preparation and immunohistochemistry

Slides of interest were digitalized by whole slide scanning (Bioimagene, Ventana Medical Systems). Five μm sections were cut from respective formalin-fixed paraffin embedded specimens for fluorescence imaging, as stated above and immunohistochemistry (IHC). IHC was performed to evaluate: EGFR (anti-human EGFR Ab-10, (1:100) Thermoscientific, Waltham, MA); density of carcinoma cells (anti-pan Cytokeratin Ab-961, (1:100) Abcam, Cambridge, MA); blood vessel density (anti-factor VIII-R, (1:100) Cellmarque, Rocklin, CA) and tumor proliferation (anti-Ki67 clone SP6, (1:100) Thermoscientific, Waltham, MA). Briefly, samples were de-paraffinized with EZ-DEWAX bath two times for 5 min. Antigen retrieval was achieved by heating for 10 min at 100 °C then cooled at room temperature and blocked with 5% BSA (Bovine Serum Albumin) in TBS (Tris Buffered Saline) for 5 min at room temperature. Slides were incubated with the primary antibodies per manufacturer recommendations. Secondary antibody (goat anti-mouse, for EGFR and cytokeratin, and goat anti rabbit, for Ki67 and Factor VIII; 1:100) was applied for 1 h in a humidified chamber at room temperature. Peroxidase activity was visualized with 3,3-diaminobenzidine (DAB+) and mounted with mounting medium and coverslipped.

### Analysis of Immunohistochemistry

Six regions ROIs from SCC and healthy surrounding tissue were selected. Staining intensities and distributions for Ki67, EGFR, cytokeratin and Factor VIII in each of the six ROIs were acquired using SPOT camera on an Olympus 1 × 70 microscope (Olympus Optical Co.), interfaced with a personal computer and SPOT software (Olympus Optical Co.). Positively staining areas were segmented by the signal intensity difference between the target cells and background in each photograph (x20), whereas the intensity and minimum particle size thresholds were set manually. Next, antigen stained cells were counted and the amount of cells per field of view (x20) reported. The ratio of EGFR, Ki67, cytokeratin and factor VIII stained areas related to the total area of specimen were calculated (EGFR-stained area/total area; Ki67-stained area/total area; cytokeratin-stained area/total area; factor VIII-stained area/total area) representing EGFR expression, cell proliferation, tumor and vascular density, respectively as described previously by us^20^. Image analysis was conducted by using ImageJ.

### Signal-to-tumor ratio for determination of margin distance to tumor

All tumor containing slides from each of the three dose groups were tested for the following inclusion criteria; i) tumor size of at least 5 mm, as determined by a board-certified pathologist, ii) intact tissue (i.e. no cuts, tears and minimal presence of adipose fat tissue), and iii) normal adjacent tissue of at least 5 mm. A ROI (3 × 1 mm) was drawn at the tumor edge with subsequent ROIs placed 1 mm situated out of the previous region. Equal sized ROIs, distant from the tumor site, were used to calculate background fluorescence intensities for each patient and subsequently subtracted from the MFI determined from each ROI. The MFI of adjacent normal tissue (i.e. 1–5 mm) was divided by the MFI of the tumor edge to determine the signal-to-tumor ratio at the predefined distances.

### Statistical analysis

Descriptive statistics were calculated for variables of interest. Statistical differences between SCC, normal tissues in terms of MFI were tested separately at each of the three cetuximab-IRDye800CW dosage levels. Mixed modeling was performed using the lme2 package in R v 3.0.1, adjusting for the number of repeated measurements per patient sample. Univariate and multivariate analysis was performed using SPSS 21.0 to separate out independent variables. Pearson correlation was performed to measure the strength of the linear relationships. Statistical significance was set at *P* ≤ 0.05.

## Additional Information

**How to cite this article**: de Boer, E. *et al*. *In Vivo* Fluorescence Immunohistochemistry: Localization of Fluorescently Labeled Cetuximab in Squamous Cell Carcinomas. *Sci. Rep.* doi: 10.1038/srep10169 (2015).

## Supplementary Material

Supplementary Information

## Figures and Tables

**Table 1:** Mean Fluorescence intensity in univariate and multivariate analysis.

**Mean Fluorescence Intensity**						
		***Univariate***			***Multivariate***	
		**R Square**	**95% CI**	**P (*****n***)	**95% CI**	**P**
2.5 mg/m^2^	Cytokeratin	0.282	1.1 - 3.4	**<0.001** (*44*)	−0.5 - 2.3	0.211
	EGFR-Density	0.526	1.6 - 3.1	**<0.001** (*40*)	1.1 - 3.2	**<0.001**
	Factor-VIII	0.090	−2.2 - 25.8	**0.048** (*32*)	−1.9 - 18.9	0.106
	Ki67	0.158	0.1 - 0.3	**0.007** (*37*)	-0.2 - 0.1	0.435
25 mg/m^2^	Cytokeratin	0.217	5.1 - 19.1	**<0.001** (*46*)	-6.6 - 10.8	0.619
	EGFR-Density	0.525	13.8 - 25.6	**<0.001** (*43*)	8.6 - 26.7	<0.001
	Factor-VIII	0.144	4.2 - 51.2	**0.011** (*36*)	−8.2 - 37.5	0.200
	Ki67	0.027	−0.3 - 0.7	0.165 (*37*)	−0.7 - 0.3	0.340
62.5 mg/m^2^	Cytokeratin	0.291	9.6 - 26.6	**<0.001** (*47*)	−7.1 - 24.4	0.255
	EGFR-Density	0.422	9.0 - 20.9	**<0.001** (*38*)	−15.5 - 27.3	0.559
	Factor-VIII	0.388	43.2 - 90.9	**0.003** (*28*)	−11.1 - 20.9	0.058
	Ki67	0.058	−0.1 - 0.5	0.105 (*29*)	−0.5 - 1.4	0.362

The ratio of EGFR, Ki67, cytokeratin and factor VIII stained areas related to the total area of specimen. *Uni- and multivariate regression analysis.*
