# Supplementary material for: In Vivo Fluorescence Immunohistochemistry: Localization of Fluorescently Labeled Cetuximab in Squamous Cell Carcinomas
Source: Sci Rep. 2015 Jun 29;5:10169. doi: 10.1038/srep10169 (PMC4894408; doi:10.1038/srep10169)
Supplement: Supplementary Information [file srep10169-s1.pdf]

In Vivo Fluorescence Immunohistochemistry: Localization of Fluorescently Labeled Cetuximab in Squamous Cell Carcinomas

Esther de Boer – Jason M. Warram – Matthew D. Tucker – Yolanda E. Hartman – Lindsay S. Moore – Johannes S. de Jong – Thomas K. Chung – Melissa L. Korb – Kurt R. Zinn – Gooitzen M. van Dam – Eben L. Rosenthal[*,Ψ] – Margaret S. Brandwein-Gensler[Ψ]

Supplementary Figure 1. Fairly reduced cetuximab-IRDye800CW uptake in mature, keratinizing tumor regions. Representative haemotoxylin/eosin (H&E), EGFR expression stain and corresponding fluorescence image of keratinizing (enclosed by black arrows) and non-keratinizing (enclosed by white arrows) tumor. Scale bars in all images represent 100 μm.

Supplementary Figure 2. Non-specific cetuximab-IRDye800CW uptake. Representative haemotoxylin/eosin (H&E) of skin, sebaceous, submandibular and sublingual gland showing co-localization of EGFR expression staining and cetuximab-IRDye800CW fluorescence. Scale bars represent: skin 200 μm; sebaceous gland 500 μm; submandibular gland 50 μm; sublingual gland 50 μm.

Supplementary Figure 1

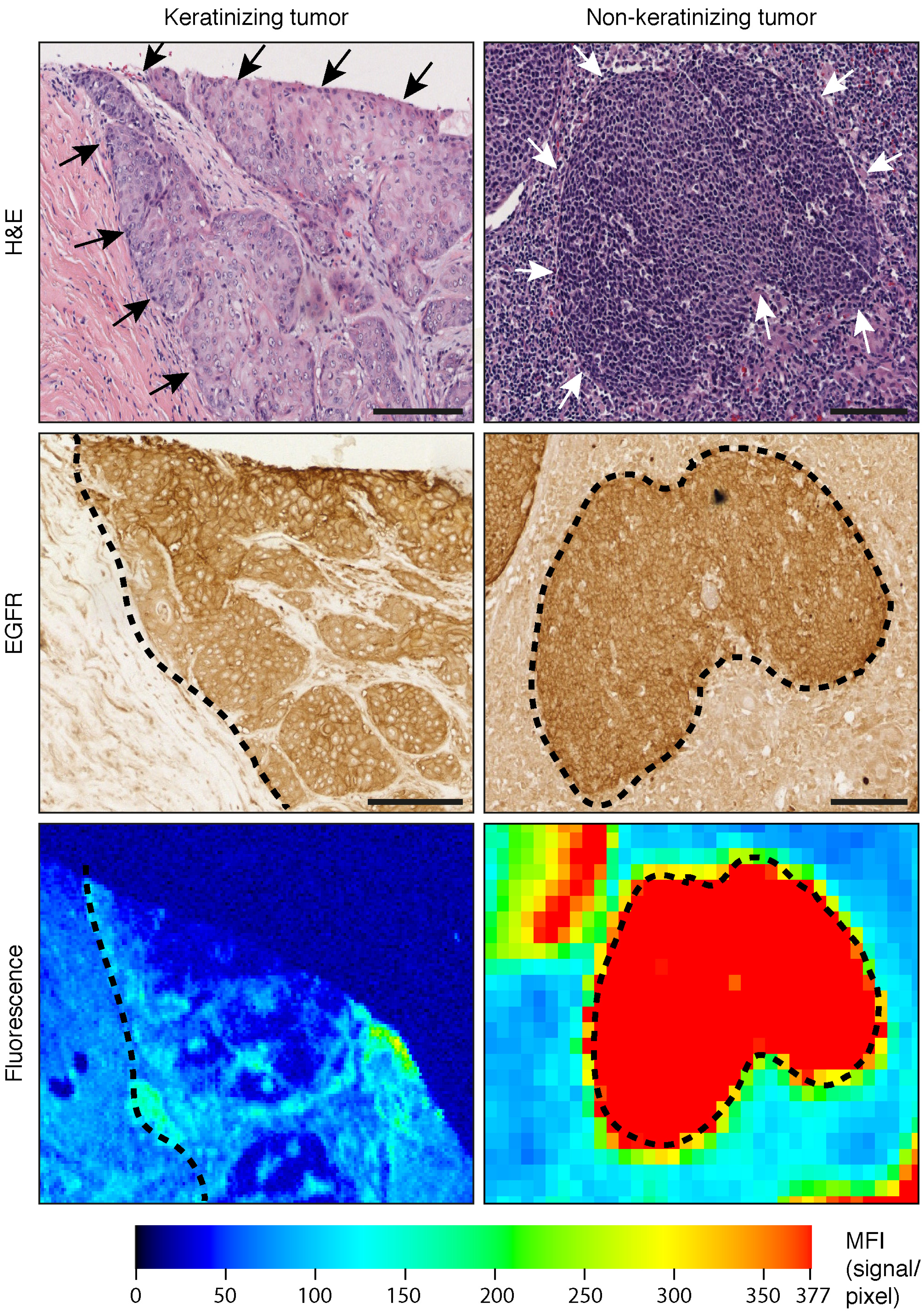

Keratinizing tumor
Non-keratinizing tumor
H&E
EGFR
Fluorescence
0
50
100
150
200
250
300
350
377
MFI
(signal/
pixel)

Supplementary Figure 2

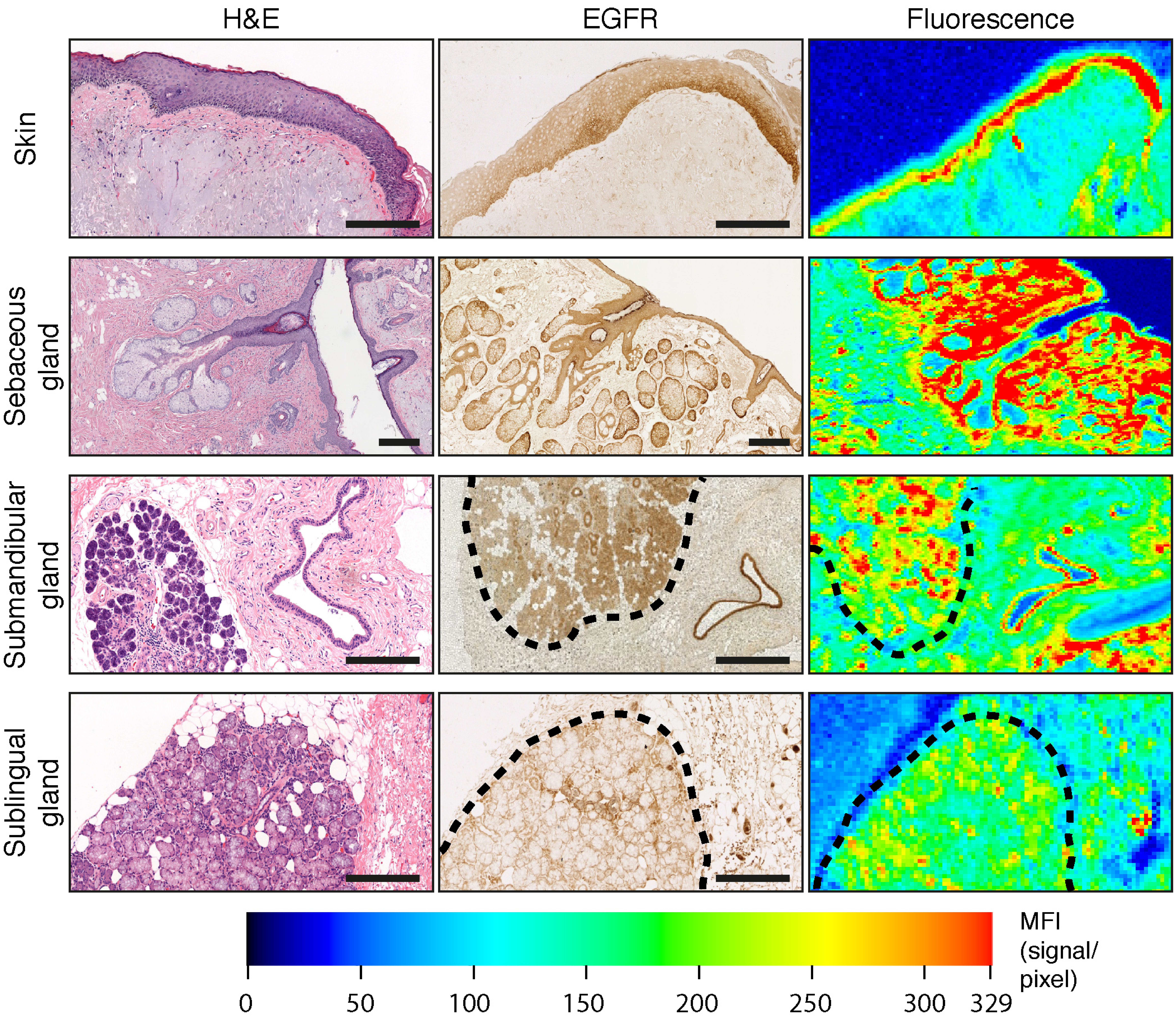
H&E
EGFR
Fluorescence
Skin
Sebaceous gland
Submandibular gland
Sublingual gland
0
50
100
150
200
250
300
329
MFI (signal/ pixel)
